# A Frame-Shift Mutation in CAV1 Is Associated with a Severe Neonatal Progeroid and Lipodystrophy Syndrome

**DOI:** 10.1371/journal.pone.0131797

**Published:** 2015-07-15

**Authors:** Isabelle Schrauwen, Szabolcs Szelinger, Ashley L. Siniard, Ahmet Kurdoglu, Jason J. Corneveaux, Ivana Malenica, Ryan Richholt, Guy Van Camp, Matt De Both, Shanker Swaminathan, Mari Turk, Keri Ramsey, David W. Craig, Vinodh Narayanan, Matthew J. Huentelman

**Affiliations:** 1 Center for Rare Childhood Disorders, Translational Genomics Research Institute, Phoenix, AZ, United States of America; 2 Neurogenomics Division, Translational Genomics Research Institute, Phoenix, AZ, United States of America; 3 Department of Medical Genetics, University of Antwerp, Antwerp, Belgium; Charité Universitätsmedizin Berlin, NeuroCure Clinical Research Center, GERMANY

## Abstract

A 3-year-old female patient presenting with an unknown syndrome of a neonatal progeroid appearance, lipodystrophy, pulmonary hypertension, cutis marmorata, feeding disorder and failure to thrive was investigated by whole-genome sequencing. This revealed a *de novo*, heterozygous, frame-shift mutation in the Caveolin1 gene (*CAV1*) (p.Phe160X). Mutations in *CAV1*, encoding the main component of the caveolae in plasma membranes, cause Berardinelli-Seip congenital lipodystrophy type 3 (BSCL). Although BSCL is recessive, heterozygous carriers either show a reduced phenotype of partial lipodystrophy, pulmonary hypertension, or no phenotype. To investigate the pathogenic mechanisms underlying this syndrome in more depth, we performed next generation RNA sequencing of peripheral blood, which showed several dysregulated pathways in the patient that might be related to the phenotypic progeroid features (apoptosis, DNA repair/replication, mitochondrial). Secondly, we found a significant down-regulation of known Cav1 interaction partners, verifying the dysfunction of *CAV1*. Other known progeroid genes and lipodystrophy genes were also dysregulated. Next, western blotting of lysates of cultured fibroblasts showed that the patient shows a significantly decreased expression of wild-type *CAV1* protein, demonstrating a loss-of-function mutation, though her phenotype is more severe that other heterozygotes with similar mutations. This phenotypic variety could be explained by differences in genetic background. Indications for this are supported by additional rare variants we found in *AGPAT2* and *LPIN1* lipodystrophy genes. *CAV1*, *AGPAT2* and *LPIN1* all play an important role in triacylglycerol (TAG) biosynthesis in adipose tissue, and the defective function in different parts of this pathway, though not all to the same extend, could contribute to a more severe lipoatrophic phenotype in this patient. In conclusion, we report, for the first time, an association of CAV1 dysfunction with a syndrome of severe premature aging and lipodystrophy. This may contribute to a better understanding of the aging process and pathogenic mechanisms that contribute to premature aging.

## Introduction

Human progeroid syndromes are a group of disorders that present some clinical features mimicking physiological aging at an early age. These syndromes are clinically and genetically heterogeneous and most, including Werner syndrome and Hutchinson–Gilford progeria, are known as segmental aging syndromes, as they do not feature all aspects associated to physiological aging [1]. At the molecular level, aging has been associated with increased oxidative stress, telomere attrition and a decrease in DNA repair mechanisms [2]. Progeroid disorders are usually monogenic, and mutations lead to the accelerated appearance of features of senescence.

Caveolin 1, encoded by the *CAV1* gene, is a highly conserved integral membrane protein that is the key component of plasma membrane invaginations known as caveolae [3]. Caveolae are important in numerous cellular functions, including signal transduction, cellular metabolism, vesicle trafficking, cholesterol homeostasis, endothelial transcytosis, and tumor suppression. In addition, Caveolin 1 has a key role in the induction of cellular senescence [3]. Caveolae are present in most cell types, but they are most conspicuous in adipocytes where they represent up to 20% of the total plasma membrane surface area.

A homozygous nonsense mutation in *CAV1* has been associated with Berardinelli-Seip congenital lipodystrophy (BSCL) or congenital generalized lipodystrophy type 3 (CGL3) (MIM# 612526), a rare syndrome characterized by the absence of adipose tissue from birth or early infancy, resulting in severe dyslipidemia, insulin resistance, and muscular hypertrophy. Mutations in *CAV1* are a rare cause of CGL; mutations in *BSCL2* (CGL2; MIM# 269700) or *AGPAT2* (CGL1; MIM# 608594) account for about 95% of reported BSCL cases [4]. In addition, mutations in and *PTRF* also cause CGL4 (MIM# 613327) [5]. Heterozygous carriers of *CAV1* mutations may or may not have a distinctive phenotype. Heterozygous carriers do not have obvious clinical features[4], or present an atypical partial lipodystrophy [6], or pulmonary hypertension [7]. Cav1-/- knockout mice show progressive lipodystrophy, insulin resistance and pulmonary hypertension and characteristics of dilated cardiomyopathy [8]^,^[9].

Children born with rare, inherited, undiagnosed conditions may benefit from a diagnosis obtained by genetic sequencing. Here, we investigated a young female patient and her unaffected parents presenting with an unknown disorder of progeroid appearance, lipodystrophy, cutis marmorata, feeding disorder and failure to thrive by genome sequencing to identify the genetic cause that causes the disease.

## Material and Methods

### Sample Collection and Ethics

We would like to thank the family for participating in this study and for allowing us to share their clinical story. The participating family provided written consent and was enrolled into the Center For Rare Childhood Disorders Program at the Translational Genomics Research Institute (TGEN). The patient was under six years of age at the time of enrollment and verbal assent was not required according to Western Institutional Review Board (WIRB). Written consent for the minor under the age of 18 years was obtained from the parents. All additional participants over 18 years of age provided written consent at the time of enrollment. The study protocol and consent procedure was approved by the Western Institutional Review Board (WIRB) (study number 20120789). The parents have given written informed consent (as outlined in PLOS consent form) to publish these case details of their child. Whole blood was collected from the proband and parents for DNA and RNA extraction. DNA was extracted using the QiaAmp blood kit and RNA was extracted using the PaxGene blood RNA system (Qiagen, Venlo, NL). RNA was also collected from 4 control samples (2 male and 2 female, mean age 46.5).

### Genome Sequencing and RNA-Seq

Genomic libraries were prepared with the Illumina’s Truseq DNA Sample Preparation Kit, following the manufacturer’s protocol. To assess the transcriptional profile of each collected RNA sample (case and 4 controls), we used Illumina’s Truseq RNA Sample Preparation Kit. Sequencing was performed by 100bp (DNA) or 50bp (RNA) paired-end sequencing on a HiSeq2000 instrument (Illumina Inc, San Diego, USA).

### Data-Analysis

Filtered reads were aligned to the Human genome (Hg19/GRC37) using the Burrows-Wheeler transform (BWA v.0.7.5) [10]. PCR duplicates were removed using Picard v1.92 [11] and base quality recalibration, indel realignment and SNP and indel discovery were performed using the Genome Analysis Toolkit (GATK v2.5–2).[12] Data was filtered against dbSNP137, 1000 Genomes, an in-house exome database, and then annotated with SnpEff 3.2a against Ensembl v66 to identify novel damaging mutations.

For RNA-seq, filtered reads were mapped to Hg19/GRC37with TopHat2 (v2.0.8) [13]. Next, Cufflinks [14] was used, which uses the aligned reads to assemble them into transcripts, estimate their abundances, and tests for differential expression. Finally, CummeRbund was used with R.2.15.3 to visualize Cuffdiff output [15]. The Reactome database was used for pathway analysis, using over-representation analysis [16]. Genes on the Y and X chromosomes were excluded due to gender differences.

### Sanger Sequencing

Primers surrounding the identified variants were designed using Primer3. Polymerase chain reaction (PCR) was carried out under standard conditions. Direct sequencing of the PCR product was performed on an ABI3130XL sequencer (Applied Biosystems Inc., Foster City, USA). For transcript analysis, RNA was transcribed into cDNA with primers targeting *CAV1* in a one step reaction (superscript III one step RT PCR kit) prior to sequencing.

### Primary Fibroblasts

3mm skin biopsy punches were obtained from the case and a random control with no distinguishable phenotype. A fibroblast cell line was established by following a modified procedure of the protocol described in Villegas et al 2005 [17]. Cells were cultured in Minimal Essential Media (Invitrogen, CA, USA) with 18% FBS (ATCC, VA, USA), Pen/Strep and Amphoteracin (Sigma-Aldrich, MO, USA) and Plasmocin (Invivogen, CA, USA).

### Western Blot

Fibroblast cells were grown on 6-well plates until 90% density and lysed using RIPA buffer in triplicate (Pierce Biotechnology, Inc., IL, USA) including a Halt protease and Phosphatase inhibitor cocktail (Thermo Scientific, MA, USA). The BCA Protein assay was used next to quantify the total protein (Thermo Scientific, MA, USA), and 15 ug of each of the 3 protein lysates was used to enter the Western Blotting procedure. In short, samples were denatured for 10 min at 95C in a sample-reducing reagent (NuPAGE; Life Technologies, Carlsbad, CA). Next, samples were loaded onto a 4–12% NuPage Bis-Tris gel in addition to a Bio-Rad Precision Plus Protein Kaleidoscope ladder (Bio-Rad, CA, USA), and ran at 1.5 hours at 125V. Next, samples were transferred onto a nitrocellulose membrane for 3h at 30V. The membrane was blocked in 5% BSA for 1h at RT, followed by overnight incubation with the primary antibody in 2.5% BSA: C-terminal rabbit anti-Cav1 antibody (ORB47981; 1/1000; Biorbyt, CA, USA) and internal epitope goat anti-Cav1 antibody (LS-B192; 1/1000; LSBio, WA, USA). Next, the secondary antibodies were incubated for 1h at RT in 2.5% BSA: goat anti-rabbit-HRP (12348; 1/5000; Cell Signaling Techn., MA, USA) and g0208 donkey anti-goat-HRP (1/5000). As a loading control: Ab20272 mouse ant beta-actin-HRP (1/5000) was used (Abcam, CA, UK). Steps were separated by washing steps with TBST. The membrane was then incubated with for 5 minutes with ECL mixture (Pierce ECL western blotting substrate) and visualized using SRX-101A processor (Konica Minolta Sensing Americas, Inc, NJ, USA). Differences in expression were calculated using LI-COR image studio Lite (LI-COR Biosciences, Nebraska, USA). Beta-actin was analyzed on the same lane in the blot as it is a different molecular weight than CAV1.

### Immunofluorescence

Fibroblast cells were fixed with 4% formaldehyde in PBS for 20 min (Thermoscientific, Waltham, MA) and blocked in 5% BSA, and 5% Donkey and Chicken serum in PBS at room temperature for 30 min before the first primary antibody (in 1% BSA) was applied for 1h at RT (Internal epitope goat anti-Cav1 antibody (LS-B192; 1/100) and mouse anti-cavin (PTRF; 1/50; SAB1408474)). Next, the secondary antibodies, Alexa Fluor 488 chicken anti-rabbit IgG and Alexa Fluor 405 Goat Anti-Mouse IgG, were applied for 1h at RT (1/2000; Life Technologies, Carlsbad, CA). All steps were separated by 3 wash steps using PBST. Coverslips were then mounted on a coverglass and visualized using a confocal microscope (ZEISS microscopy, Oberkochen, Germany) using 40X magnification, and analyzed using ZEN Microscope software 2012.

## Results

### Clinical Representation

This is a 3 year-old girl with Caucasian ethnicity with severe lipoatrophy, pulmonary hypertension, and a progeroid appearance (Fig A in S1 File). Amniocentesis was performed during this pregnancy and showed a normal fetal karyotype. Fetal ultrasound revealed bilateral pleural cavity fluid collections, thought to represent chylothorax. These resolved by the 7th or 8th month of gestation. Fetal growth rate was slow.

Delivery was uncomplicated, by repeat C-section at term, and no fetal resuscitation was required. A chest X-ray was done for tachypnea, which revealed bilateral small pneumothoraces. This resolved spontaneously. As a neonate, she had a poor suck, and cried inconsolably while she nursed.

As an infant, skin findings noted on her exam included a fine reticular pattern of her skin (livedoreticularis or cutis marmorata), large anterior fontanelle, absence of subcutaneous fat, with a history of feeding problems, vomiting and failure to thrive. She was given N-G tube feedings of breast milk. Weight gain remained poor, and she did not tolerate greater than 1-oz bolus feedings. She continued to have daily episodes of projectile vomiting, in spite of thickened feedings. At 8 months of age, a feeding gastrostomy was placed and she was fed continuously. She presented with hypertriglyceridemia at age 3 months, and has had fluctuating episodes of elevated triglyceride levels and normal levels since her first test.

Her development has been normal. She did not have seizures and was always active. Initial Neurological Examination at 7 months found that she was thin, her weight well below 5%, and her height and head circumference were between 60–75%. She had very little subcutaneous fat, livedoreticularis of the skin, prominent skin veins, a triangular appearance to her face, and a large anterior fontanelle, extending into the metopic suture.

Subsequent examinations continued to show lack of subcutaneous fat, livedoreticularis, large and open anterior fontanelle, but no focal neurological findings. She was active, playful, interactive, and was speaking in sentences. She developed echocardiographic features of pulmonary hypertension, confirmed by catheterization. Ophthalmological examination was normal.

Brain MRI done at 3 months and 8 months showed prominent subarachnoid spaces, ventricles, and Sylvian fissure, raising the possibility of cerebral atrophy. Upper GI studies were normal. Thyroid function tests, complete metabolic profile, plasma amino acids, urine organic acids, and array CGH were normal. Bone age was normal; skull X-ray showed only the large anterior fontanelle, but absence of Wormian bones. Genetic testing for Russell-Silver syndrome, mandibulo acral dysplasia were negative. Sweat chloride test was normal, and a chest CT scan was normal. Lung biopsy showed focal smooth muscle thickening of pre-acinar arterioles. At 4-years old, the patient’s fasting serum leptin was 0.7 ng/mL, which is much lower than healthy 4-year old females: 1.9 (1.5–2.7) [18].

### Genome Sequencing

Average genome sequencing depth was 39x, with 96.93% over 10x depth. Variant calling revealed a de novo frame-shift mutation in the *CAV1* gene (chr7:116199282–3>delTT; NM_001172897.1:c.385_386delTT;NP_001166368.1:p.Phe129X; NM_001753.4:c.478_479delTT;NP_001744.2:p.Phe160X), previously associated with Congenital generalized lipodystrophy type 3. The mutation introduces an immediate stop into the protein. Whole genome sequencing could not reveal a second mutation in *CAV1*, although coverage across all exons was sufficient to detect variations. In addition, we also found a second variant of interest in a gene associated with congenital generalized lipodystrophy type 1: *AGPAT2* (chr9:139581758insCAG; NM_006412.3:c.51_52insGTC; NP_006403.2:p.X18Leu; rs201504151). The variant in *AGPAT2* is inherited from the father, though quite frequently present in the normal population (7.18%, exome variant server). It is unclear whether this variant contributes to any symptoms present the patient. A last interesting variant was found in lipin 1 (*LPIN1*), in which mutations cause a lipoatrophic phenotype in the mouse [19] (chr2: 11927238G>A;NP_001248356.1:p.Val500Met; rs33997857). This variant has a 2.2% frequency in the population, was inherited from the mother, and is predicted damaging. The CADD scores and details of all 3 variants are listed in Table 1 [20]. All variants were confirmed with Sanger sequencing in the patient and parents. Studying mRNA extracted from the patient’s whole blood revealed that both mutant and wild type CAV1 were transcribed (Fig B in S1 File), and that the RNA from the mutant allele was neither degraded by nonsense mediated RNA decay; nor was the second allele silenced by a regulatory mutation.

**Table 1:** Genetic variants identified in the patient.

Chr	Pos	ID	Ref	Alt	Protein change	Gene	CADD score	MAF EVS
2	11927238	rs33997857	G	A	NP_001248356.1:p.Val500Met	LPIN1	21.6	1.63%
**7**	**116199281**	**.**	**CTT**	**C**	**NP_001744.2:p.Phe160X**	**CAV1**	**29.5**	**none**
9	139581758	rs201504151	C	CCAG	NP_006403.2:p.X18Leu	AGPAT2	10.75	7.20%

In bold is the mutation in CAV1. MAF EVS = Minor allele frequency Exome variant server (all populations).

### RNA-Seq Differential Expression Analysis

#### Pathway analysis

**Fig 1:**
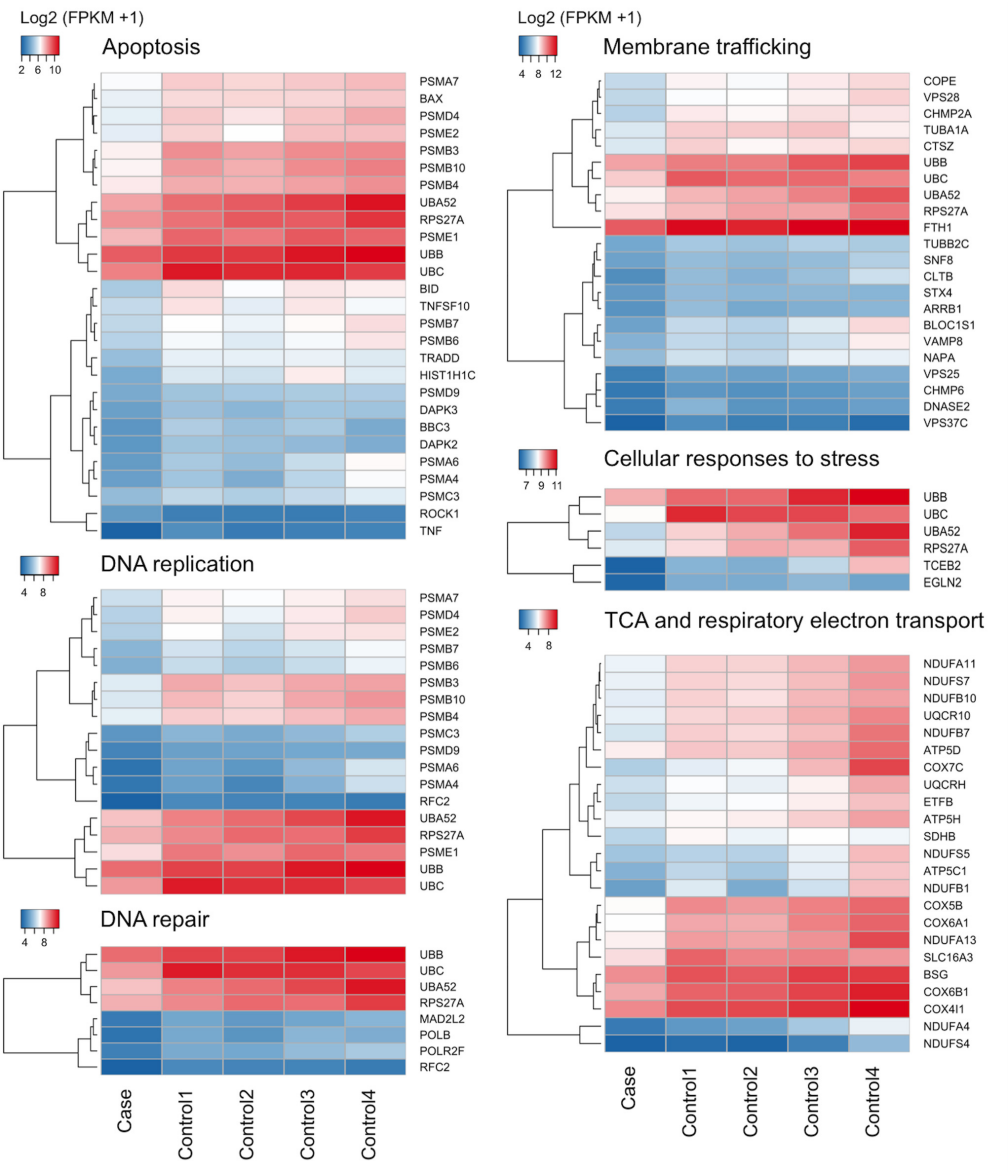
Expression heatmaps of RNA analyzed from whole blood. All genes are displayed that reach significance after correction for multiple testing FDR and have an FPKM above 1. Interesting dysregulated pathways and subpathways are: Regulation of apoptosis (p = 3.0 x 10–8), DNA replication, with affected sub-pathways regulation of DNA replication (p = 1.4 x 10–5), M/G1 transition (p = 5.2 x 10–5) and the synthesis of DNA (p = 2.1 x 10–4)). DNA repair is also dysregulated, especially the fanconi anemia pathway (Translocation of ub-FANCD2 and ub-FANCI to chromatin; p = 4.0 x 10–4 and regulation of the Fanconi anemia pathway 4.4 x 10–3). Also membrane trafficking is affected, especially subpathway Endosomal Sorting Complex Required For Transport (ESCRT) (p = 1.8 x10-5). The citric acid (TCA) cycle and respiratory electron transport pathway is significantly dysregulated (p = 9.3x10-6) and cellular responses to stress as well (p = 8.6 x 10–3). For signal transduction, especially signaling by Wnt is affected (p = 3.2 x 10–4).

Overrepresentation analysis was done to identify pathways that are dysregulated in our patient compared to 4 healthy controls, unaffected relatives from patients of our rare childhood disorder center [16]. 1346 genes reached significance after multiple testing (FDR correction) (2.5% of total genes), 519 were recognized in a REACTOME pathway for analysis (Table A in S1 File). Most genes were found to be downregulated. Most significantly dysregulated pathways are: Translational regulation, RNA metabolism and protein metabolism and most interesting pathways are shown in Fig 1.

#### Dysregulation of Progeroid Syndrome and Lipodystrophy Genes

**Fig 2:**
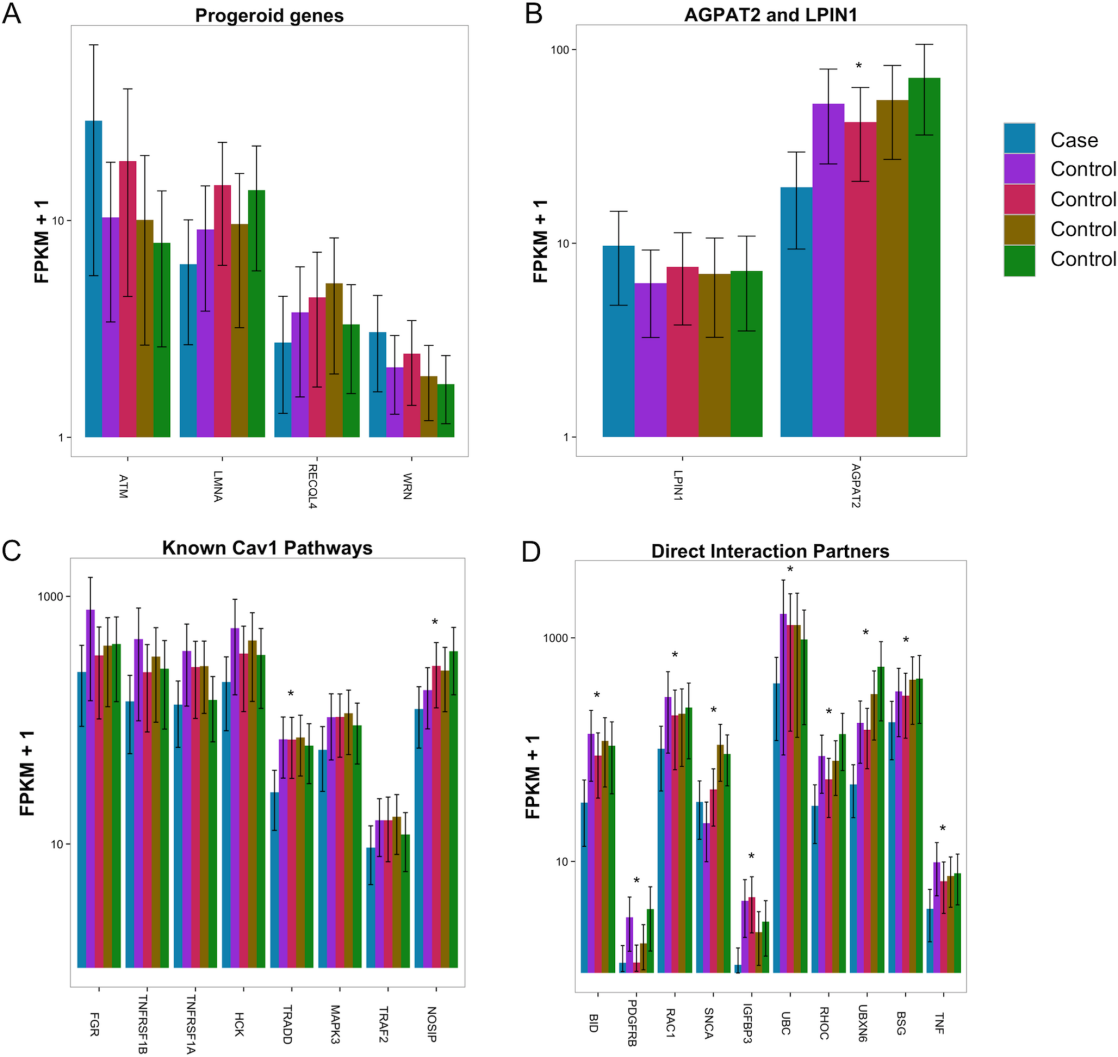
A. Expression bar plots of RNA analyzed from whole blood involved in other progeroid syndromes. B. AGPAT2, lipodystrophy. C. Known Cav1 pathways and D. Direct interaction partners. * Reach significance after correction for multiple testing (FDR). LPIN1 is not significantly different between case and controls. 95% FPKM cufflinks confidence intervals for estimates were obtained using a Bayesian inference method based on importance sampling from the posterior distribution [21].

In addition, we looked at genes involved in other progeroid and lipodystrophy syndromes (Fig 2). We found that several progeroid syndrome genes are dysregulated: *LMNA*, *ATM*, *RECQL4* and *WRN*. The gene causing Berardinelli-Seip Congenital Lipodystrophy (*AGPAT2*) is very significantly underexpressed (padj = 0.002; Fig 2). *BSCL2*, *PTRF* and *LPIN1* lipodystrophy genes are not differentially expressed.

#### Deregulation of Cav1 Direct Interaction Partners and Downstream Pathways

129 proteins are found to interact directly with Cav1 based previous studies (NCBI gene interactions; http://www.ncbi.nlm.nih.gov/gene), and 102 of these were expressed highly enough in RNA extracted from whole blood to perform differential analysis. Of these 102, 30 were significantly different between our case and the controls (29.4%) (Table B in S1 File), and 72 (73.3%) are down regulated (Fig 2).

Caveolins interact with a variety of downstream signaling molecules, including Src-family tyrosine kinases, p42/44 mitogen activated protein (MAP) kinase, and endothelial nitric oxide synthase (eNOS) [22]. In the Caveolin downstream pathway, we found a significant under expression of NOSIP, a modulator of eNOS. *MAPK3* (p44) is underexpressed, and HCK and FGR are also significantly underexpressed (SRC-family tyrosine kinases) (Fig 2). Caveolin-1 associates with TRAF2 to form a complex that is recruited to tumor necrosis factor receptors [23]. In this pathway, *TRADD*, *TRAF2*, *TNFRSF1A* and *TNFRSF1B* are also significantly underexpressed (Fig 2).

#### Expression of CAV1 in the Proband

**Fig 3:**
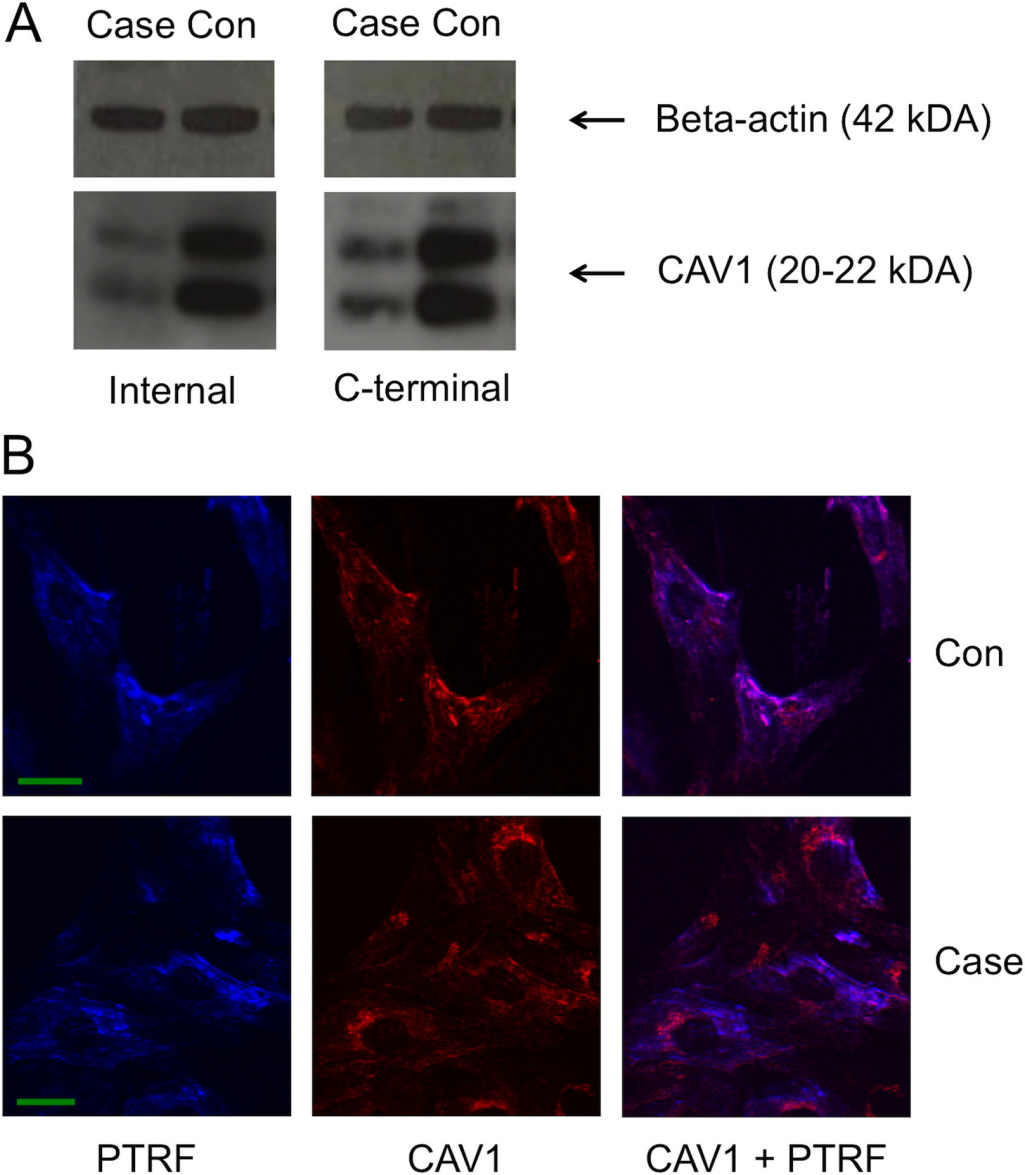
A. western blot of lysates from case and control fibroblasts. Two antibodies were used, one that recognized an internal epitope in CAV1 (LS-B192), and one that recognized a C-terminal epitope (ORB47981; would only recognize WT CAV1). Beta-actin was used as a loading control. Calculation of intensity of the CAV1 isoform bands compared to the loading control, illustrated that WT expression is decreased in the case compared to the control. B: Immunocytochemistry of case and control fibroblast cells with anti-PRTF and anti-CAV1 antibodies. In the control, co-localization of CAV1 and PTRF (Caveolae marker) is very clear. In the proband’s cells, this co-localization is less consistant. Green bar = 28um.

Protein lysates from case and control fibroblasts were subjected to western blotting to measure protein expression of CAV1 (in triplicate). Two different antibodies were used: one that targets the C-terminus of CAV1 (and would only recognize the wild type (WT) CAV1), and one that recognized an internal epitope of CAV1 (which would recognize both WT and mutant (MT) CAV1 if expressed). All blots showed a significant decrease in expression of CAV1 compared to the control that was similar between an antibody that detected WT CAV1 only and an antibody detecting both WT + MT CAV1, suggesting MT CAV1 is not expressed or expressed at a very low level (Fig 3). In addition, fibroblasts were grown on glass coverslips, fixed and stained for localization of CAV1 and PTRF (Caveolae marker) (Fig 3). In the control, co-localization of CAV1 and the caveolae marker was perfect, however, the co-localization was not entirely fully concordant in the case (Fig 3).

## Discussion

In this study, we investigated a young patient with a heterozygous *CAV1* mutation who presented with a progeroid appearance, lipodystrophy and pulmonary hypertension. Although some of these features have been seen in other *CAV1* heterozygotes, our patient presents a much more severe syndrome that includes a progeroid apperearance, which has not previously has been linked to *CAV1* mutations. These results expand the phenotypic spectra of *CAV1* associated disorders. In addition, we identified several dysregulated pathways by expression analysis that might contribute to this phenotype.

We identified a mutation in *CAV1* in our patient that truncates the last part of the protein (p.Phe160X). Heterozygotes with an early truncation in *CAV1* (p.Glu38X), do not show any phenotype [4]. p.Glu38X affects both the alpha- and beta-Cav1 isoforms and completely ablates Cav1 expression in skin fibroblasts [4]. Some heterozygotes with a -88delC (regulatory) and p.Ile134fsdelA-X137 mutation show atypical partial lipodystrophy and hypertriglyceridemia [6], and other heterozygotes with a loss-of-function p.P158HfsX22 mutation show only pulmonary arterial hypertension [7]. Since p.Phe160X mutant RNA is still expressed in our patient, we first hypothesized that the mutant protein is expressed and could act in a dominant negative fashion towards the wild type, as certain deletion/mutation constructs of Cav1 (disrupted scaffolding and COOH-terminal domains) have also been shown previously to act in a dominant-negative manner when expressed in HEK293T cells [24]. However, we showed that the proband expresses less than half of the WT protein (Fig 3), with no or very little trace of MT CAV1, suggesting this is a loss-of-function mutation. Her phenotype is however more severe that other heterozygotes with similar mutations. The phenotypic variety could be explained by differences in genetic background. We have identified two other variants that might act as modulators and aggravate her phenotype to support this hypotheses (Table 1). Mutations in the *LPIN1* gene causes a lipoatrophic phenotype in the mouse [19] and mutations in the *AGPAT2* gene are associated with congenital generalized lipodystrophy type 1 in humans and similar symptoms in mice [25] [26]. The patient carries a variant in *LPIN1* (rs33997857; Val500Met), which has a low frequency of 2.2% in the European/American population, but is highly conserved (PhyloP = 2.63). In a large German population (n = 1,674), this variant was part of a haploblock highly associated with decreased BMI and decreased waist circumference [27], however, this could not be confirmed in follow up studies [28].

The principal metabolic function of adipocytes is to synthesize triacylglycerol (TG) from exogenous fatty acids. Lipins are phosphatidate phosphatase (PAP) enzymes that catalyze the penultimate step in triacylglycerol (TAG) biosynthesis, the dephosphorylation of phosphatidate to diacylglycerol. The fact that humans with lipin-1 deficiency do not manifest lipodystrophy suggests that a compensatory mechanism for TAG synthesis could exist in human adipose tissue [29]. However, our case also carries a rare variant in *AGPAT2* (insertion of Leu at position 18), another important attenuator of triacylglycerol (TAG) biosynthesis [25]. Mutations in *AGPAT2* may cause congenital generalized lipodystrophy by inhibiting triacylglycerol synthesis and storage in adipocytes [24]. And last, triacylglycerol is synthesized in a specific subclass of caveolae in primary adipocytes [30]. The cumulative combination of the defective functions of *CAV1*, *LPIN1* and *AGPAT2* genes in TAG biosynthesis, though probably in different degrees for each gene, might explain the severe lipoatrophic phenotype in this case.

When investigating the mRNA expression profile in our patient compared to 4 healthy controls, several dysregulated pathways related to Cav1 or phenotypic characteristics were revealed. Pathway analysis showed that several significantly down regulated genes are involved in apoptosis (Fig 1). Recent accumulating evidence indicates that dysregulation of the apoptotic process may be involved in some aging processes [31]. Caveolin-1 has been shown to be important in apoptosis as it mediates Fas-BID signaling in hyperoxia-induced apoptosis [32]. BID is 3.9x downregulated in addition to other genes involved in apoptosis (Fig 1, Fig 2).

Another affected pathway is cellular responses to stress (Fig 1). Caveolin-1 has a key role in the induction of cellular senescence and over-expression of caveolin-1 in fibroblasts can induce premature senescence [3]. Also, membrane trafficking in general is dysregulated (Fig 1), since Cav1 is required to form invaginated caveola and important in membrane trafficking.

Pathophysiologically, known Progeroid syndromes are caused, either by mutations in genes encoding DNA repair proteins, or by mutations in genes encoding lamins A/C (LMNA/C) or partners involved in their biological pathway [1]. In addition, several animal models featuring premature aging have abnormal mitochondrial function or signal transduction between membrane receptors, nuclear regulatory proteins and mitochondria [1]. Our data analysis shows that DNA repair, especially the Fanconi anemia pathway, is down regulated in addition to mitochondrial citric acid (TCA) cycle and respiratory electron transport pathway (p = 9.3x10-6) (Fig 1). The Fanconi anemia pathway leads to formation of DNA repair structures [33]. Aging in man is accompanied by a decline in mitochondrial function, mainly affecting the respiratory chain. In addition to this, known genes involved in segmental progeroid syndromes are also dysregulated, including *ATM* and *LMNA* (Fig 2).

When looking at lipodystrophy genes, *AGPAT2* is significantly downregulated (Fig 2). It is unclear whether this downregulation is a consequence of lipodystrophy, or perhaps the variant found in *AGPAT2*, p.X18Leu; rs201504151, which has a frequency of 7% in the population, might contribute to this effect. Next, genes involved in white adipocyte differentiation are dysregulated as well (*RXRA; MED16; MED25*; data not shown) and direct interaction partners or other pathways linked *CAV1* all seem downregulated as well (Fig 2), which confirms the dysfunctionality of Cav1 in this patient. CAV1 dysfunction is further demonstrated by the non-perfect co-localization of CAV1 with a caveolae marker in the patient’s fibroblasts (Fig 3).

Lastly, during the revision of this manuscript, another paper was published [34], describing *de novo* heterozygous CAV1 null mutations, c.424C>T (p. Q142*) and c.479_480delTT (p.F160*), in a 7-year- old male and a 3-year-old female of European origin with neonatal onset of generalized loss of subcutaneous fat and progeroid features. While we cannot be fully certain, the 3-year-old female patient described in Garg *et al*. is suspected to be the same patient as we describe in this manuscript. We find this result encouraging for the field as two different groups performed exome sequencing and analysis of the same patient and reached the same conclusion regarding the genetic basis of disease. This is impressive as exome sequencing and analysis approaches can differ dramatically and the prioritization of the final resulting variant collection is even more individualized across laboratory groups. Additionally, the follow-up *in vitro* examination of the variant’s impact on CAV1 resulted in similar findings. Garg *et al*. demonstrate a decrease in CAV1 protein expression, as do we, and they could not find any differences in the number and morphology of caveolae upon electron microscopy examination [34].

In conclusion, we have identified a heterozygous loss-of-function mutation in *CAV1* in a young patient with a neonatal lipodystrophy and progeroid syndrome, and identified dysregulated pathways that might be important in the contribution to her phenotypic features, including apoptosis, DNA replication, DNA repair, cellular responses to stress, mitochondrial TCA and respiratory electron transport. In addition, 29.4% of known Cav1 interaction partners were downregulated, verifying the dysfunction of *CAV1*. Cav1 mutations carriers seem to cause a phenotypic spectra ranging from no symptoms to pulmonary hypertension, partial lipodystrophy and a severe progeroid syndrome. We suggest that genetic background might contribute to this phenotypical variation in disease severity.

## Supporting Information

S1 File Supplementary information. **(Fig A).** A picture of the proband **(Fig B).** Cav1 RNA transcript analysis. Sanger sequencing of PCR products using transcript specific primers. The affected child expresses both WT and MT Cav1 **(Table A).** Significantly dysregulated pathways in the patient compared to controls (Source: RNA extracted from whole blood) **(Table B).** Significantly dysregulated genes in the patient compared to controls (source: RNA extracted from whole blood).
(DOCX)
