# Supplementary material for: A Frame-Shift Mutation in CAV1 Is Associated with a Severe Neonatal Progeroid and Lipodystrophy Syndrome
Source: PLoS One. 2015 Jul 15;10(7):e0131797. doi: 10.1371/journal.pone.0131797 (PMC4503302; doi:10.1371/journal.pone.0131797)
Supplement: S1 File — (Fig A). A picture of the proband (Fig B). Cav1 RNA transcript analysis. Sanger sequencing of PCR products using transcript specific primers. The affected child expresses both WT and MT Cav1 (Table A). Significantly dysregulated pathways in the patient compared to controls (Source: RNA extracted from whole blood) (Table B). Significantly dysregulated genes in the patient compared to controls (source: RNA extracted from whole blood). (DOCX) [file pone.0131797.s001.docx]

**Supplementary Data**

**Figure A:** A picture of the proband

**
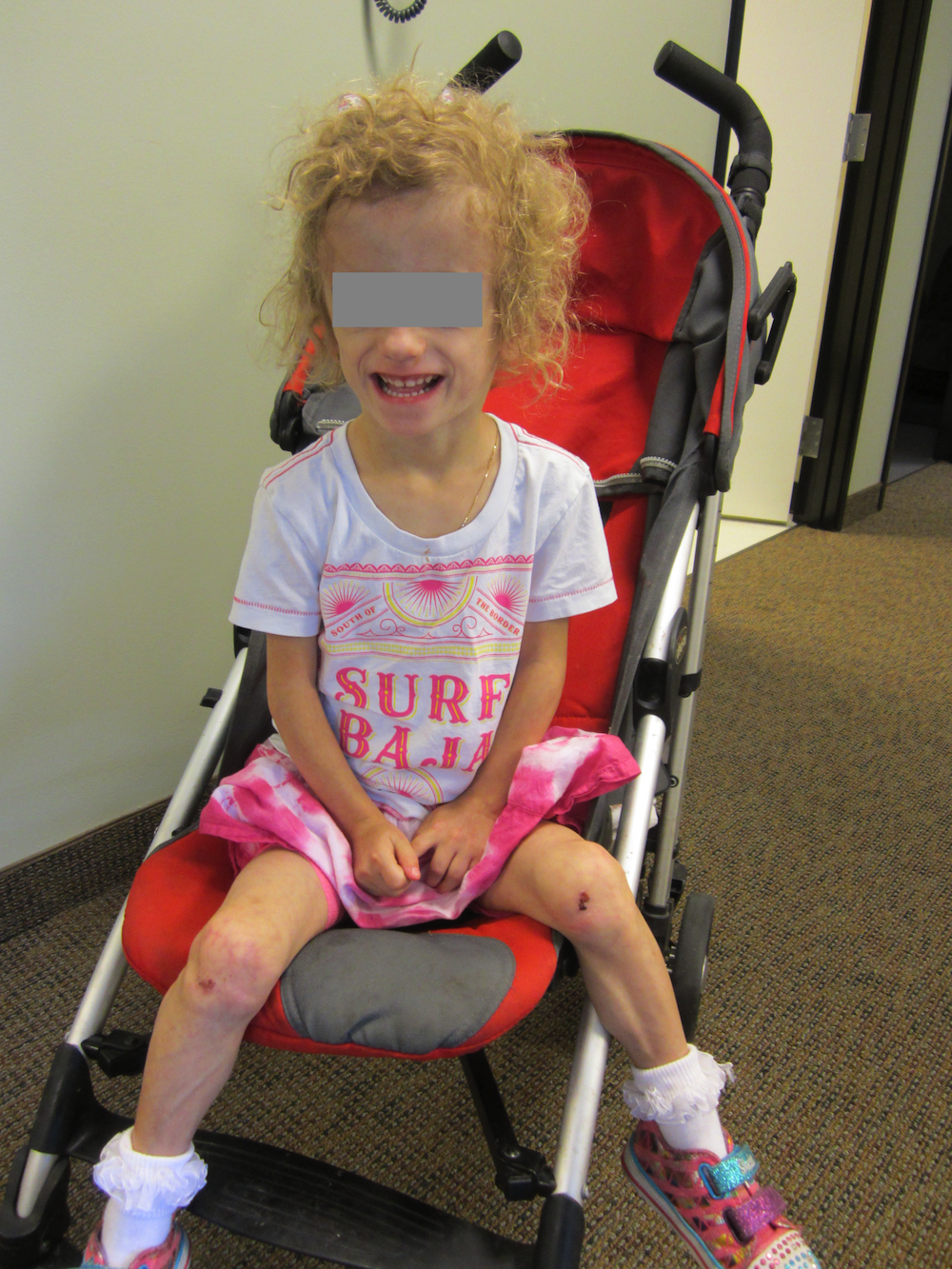
**

**Figure B:** Cav1 RNA transcript analysis. Sanger sequencing of PCR products using transcript specific primers. The affected child expresses both WT and MT Cav1.


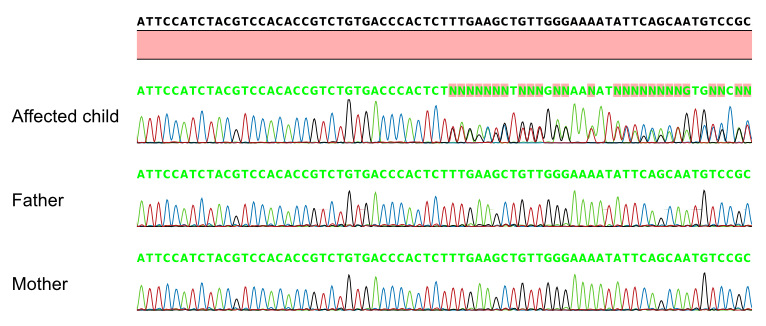


**Table A: Significantly dysregulated pathways in the patient compared to controls** (Source: RNA extracted from whole blood).

| **Pathway** | **Sub Pathway** | **p-value** | **Total in Pathway** | **Upregulated** | **Downregulated** |
| --- | --- | --- | --- | --- | --- |
| Gene Expression | | 1.00E-01 | 1031 | 4 up | 83 down |
|  | Translation | 1.00E-16 | 151 | 0 up | 45 down |
|  | Nonsense-Mediated Decay | 1.10E-16 | 110 | 0 up | 38 down |
|  | Regulation of mRNA Stability by Proteins that Bind AU-rich Elements | 4.70E-05 | 86 | 0 up | 18 down |
| Metabolism of proteins | | 5.30E-07 | 574 | 3 up | 72 down |
|  | Translation | 1.00E-16 | 151 | 0 up | 45 down |
| Disease |  | 1.80E-08 | 915 | 13 up | 99 down |
|  | Influenza Infection | 4.10E-15 | 121 | 0 up | 45 down |
| Metabolism of RNA | | 9.80E-16 | 249 | 1 up | 57 down |
| 3' -UTR-mediated translational regulation | | 1.10E-17 | 109 | 0 up | 39 down |
| Immune System | | 4.00E-06 | 1397 | 32 up | 113 down |
| Apoptosis | | 2.10E-05 | 154 | 1 up | 26 down |
|  | Regulation of Apoptosis | 3.00E-08 | 60 | 0 up | 19 down |
| DNA Replication | | 4.00E-04 | 101 | 0 up | 18 down |
|  | Regulation of DNA replication | 1.40E-05 | 72 | 0 up | 17 down |
|  | M/G1 Transition | 5.20E-05 | 79 | 0 up | 17 down |
|  | Synthesis of DNA | 2.10E-04 | 96 | 0 up | 18 down |
| Mitotic M-M/G1 phases | | 3.80E-02 | 266 | 3 up | 25 down |
|  | M/G1 Transition | 5.20E-05 | 79 | 0 up | 17 down |
|  | M Phase | 8.40E-03 | 235 | 3 up | 25 down |
| Cell Cycle |  | 4.20E-01 | 478 | 5 up | 32 down |
|  | Cell Cycle Checkpoints | 9.30E-04 | 117 | 0 up | 19 down |
| Proteasome mediated degradation of PAK-2p34 | | 3.50E-08 | 49 | 0 up | 17 down |
| Metabolism | | 5.90E-03 | 1486 | 18 up | 116 down |
|  | The citric acid (TCA) cycle and respiratory electron transport | 9.30E-06 | 131 | 0 up | 25 down |
|  | Metabolism of porphyrins | 6.90E-04 | 16 | 0 up | 6 down |
|  | Metabolism of nucleotides | 1.60E-03 | 78 | 2 up | 12 down |
| Membrane Trafficking | | 8.00E-03 | 192 | 0 up | 24 down |
|  | Endosomal Sorting Complex Required For Transport (ESCRT) | 1.80E-05 | 28 | 0 up | 10 down |
| Signal Transduction | | 1.00E+00 | 1847 | 19 up | 81 down |
|  | Signaling by Wnt | 3.20E-04 | 91 | 0 up | 17 down |
| Cdc20:Phospho-APC/C mediated degradation of Cyclin A | | 1.70E-06 | 69 | 0 up | 18 down |
| Ubiquitination of PAK-2p34 | | 1.40E-04 | 5 | 0 up | 4 down |
| TRAF6 Mediated Induction of proinflammatory cytokines | | 2.90E-01 | 60 | 0 up | 6 down |
|  | Activated TRAF6 synthesizes unanchored polyubiquitin chains upon TLR stimulation | 1.40E-04 | 5 | 0 up | 4 down |
|  | TAK1 activates NFkB by phosphorylation and activation of IKKs complex | 2.00E-02 | 22 | 0 up | 5 down |
| DNA Repair | | 2.70E-01 | 107 | 1 up | 9 down |
|  | Fanconi Anemia pathway | 9.80E-02 | 24 | 0 up | 4 down |
| Rnf111 ubiquitinates Smad7 | | 4.00E-04 | 6 | 0 up | 4 down |
| Transmembrane transport of small molecules | | 4.80E-01 | 503 | 12 up | 26 down |
|  | Iron uptake and transport | 4.20E-04 | 39 | 0 up | 10 down |
| Receptor-ligand binding initiates the second proteolytic cleavage of Notch receptor | | 1.70E-02 | 14 | 0 up | 4 down |
|  | Murine ADAM10 cleaves Notch at the S2 site producing | 8.80E-04 | 7 | 0 up | 4 down |
|  | transmembrane spanning NEXT and ligand-bound NECD |  |  |  |  |
| Circadian Clock | | 2.60E-01 | 35 | 0 up | 4 down |
|  | Ubiquitination of CRY Proteins | 8.80E-04 | 7 | 0 up | 4 down |
|  | Ubiquitination of PER Proteins | 2.80E-03 | 9 | 0 up | 4 down |
| Cellular responses to stress | | 8.60E-03 | 25 | 0 up | 6 down |
| FBXW7 mediates ubiquitination of phosphorylated NICD1 | | 2.80E-03 | 9 | 0 up | 4 down |
| SMURF2 ubiquitinates Smad7 and phosphorylated TGFBR1 | | 2.80E-03 | 9 | 0 up | 4 down |
| SMURF1 ubiquitinates Smad7 and phosphorylated TGFBR1 | | 2.80E-03 | 9 | 0 up | 4 down |
| NEDD4L ubiquitinates Smad7 and TGFBR1 | | 2.80E-03 | 9 | 0 up | 4 down |
| Uchl5 is recruited to TGF-beta receptor complex through Smad7 | | 2.80E-03 | 9 | 0 up | 4 down |
| Uchl5 deubiquitinates TGFBR1 | | 2.80E-03 | 9 | 0 up | 4 down |
| Receptor-ligand binding initiates the second proteolytic cleavage of Notch receptor | | 1.70E-02 | 14 | 0 up | 4 down |
|  | NOTCH2-ligand complex is cleaved to produce NEXT2 | 4.40E-03 | 10 | 0 up | 4 down |
|  | Complex of NOTCH1 with its ligand is cleaved to produce NEXT1 | 6.50E-03 | 11 | 0 up | 4 down |
| Hemostasis | | 1.50E-01 | 481 | 5 up | 37 down |
|  | Cell surface interactions at the vascular wall | 1.90E-02 | 93 | 2 up | 11 down |
| Ubiquitination of SKI/SKIL by Rnf11 | | 9.20E-03 | 12 | 0 up | 4 down |
| APC-Cdc20 mediated degradation of Nek2A | | 2.40E-02 | 23 | 0 up | 5 down |

**Table B: Significantly dysregulated genes in the patient compared to controls** (source: RNA extracted from whole blood).

| **Gene** | **FPKM case** | **FPKM controls** | **Fold change (log2)** | **p-value** | **q-value** |
| --- | --- | --- | --- | --- | --- |
| BID | 32.52 | 126.90 | -1.96 | 0.00005 | 0.00213 |
| KCNMA1 | 0.01 | 0.58 | -5.28 | 0.00005 | 0.00213 |
| RHOC | 30.43 | 98.88 | -1.70 | 0.00005 | 0.00213 |
| UBXN6 | 47.87 | 306.63 | -2.68 | 0.00005 | 0.00213 |
| UBC | 391.65 | 1641.98 | -2.07 | 0.00035 | 0.00924 |
| PDGFRB | 0.24 | 1.48 | -2.62 | 0.00040 | 0.01032 |
| IGFBP3 | 0.19 | 2.59 | -3.77 | 0.00060 | 0.01370 |
| Clic4 | 7.70 | 2.66 | 1.53 | 0.00120 | 0.02188 |
| TNF | 2.76 | 7.30 | -1.40 | 0.00185 | 0.02874 |
| SNCA | 33.09 | 75.16 | -1.18 | 0.00215 | 0.03183 |
| RAC1 | 101.20 | 240.45 | -1.25 | 0.00295 | 0.03872 |
| HRAS | 10.60 | 23.75 | -1.16 | 0.00325 | 0.04111 |
| BSG | 174.69 | 373.40 | -1.10 | 0.00430 | 0.04918 |
| MAPK3 | 56.65 | 114.23 | -1.01 | 0.00495 | 0.05409 |
| TNFRSF1B | 141.00 | 344.39 | -1.29 | 0.00530 | 0.05631 |
| STRN4 | 24.48 | 47.78 | -0.96 | 0.00715 | 0.06693 |
| TOLLIP | 17.75 | 37.62 | -1.08 | 0.00775 | 0.07066 |
| IRS1 | 1.18 | 0.32 | 1.88 | 0.01110 | 0.08831 |
| S1PR1 | 52.87 | 29.24 | 0.85 | 0.01180 | 0.09165 |
| TNFRSF1A | 132.86 | 266.20 | -1.00 | 0.01510 | 0.10732 |
| TRAF2 | 8.33 | 14.65 | -0.81 | 0.01790 | 0.11935 |
| STRN | 5.33 | 3.10 | 0.78 | 0.01860 | 0.12180 |
| FLOT1 | 109.07 | 198.77 | -0.87 | 0.01880 | 0.12235 |
| BMX | 4.83 | 2.52 | 0.94 | 0.02130 | 0.13295 |
| APP | 29.83 | 17.75 | 0.75 | 0.02295 | 0.13863 |
| PTPN6 | 271.10 | 571.99 | -1.08 | 0.03085 | 0.16351 |
| RCVRN | 0.09 | 0.32 | -1.77 | 0.03475 | 0.17611 |
| IRAK1 | 26.19 | 46.71 | -0.83 | 0.03665 | 0.18239 |
| LRP6 | 0.47 | 0.13 | 1.90 | 0.04135 | 0.19581 |
| TGFBR1 | 8.67 | 5.54 | 0.65 | 0.04385 | 0.20306 |
| GNAI2 | 456.51 | 848.53 | -0.89 | 0.05100 | 0.22249 |
| KCNA3 | 4.16 | 2.50 | 0.74 | 0.05170 | 0.22480 |
| FAS | 6.91 | 13.50 | -0.97 | 0.05305 | 0.22827 |
| FLOT2 | 185.55 | 302.82 | -0.71 | 0.06880 | 0.26290 |
| CXCR4 | 151.66 | 232.34 | -0.62 | 0.07070 | 0.26718 |
| BMPR2 | 2.38 | 1.32 | 0.85 | 0.07090 | 0.26755 |
| ILK | 64.58 | 123.19 | -0.93 | 0.07290 | 0.27171 |
| DAG1 | 3.87 | 2.53 | 0.61 | 0.07875 | 0.28356 |
| SMAD2 | 6.14 | 4.18 | 0.56 | 0.07915 | 0.28443 |
| PPP2R1A | 122.55 | 191.72 | -0.65 | 0.08040 | 0.28676 |
| CSK | 169.49 | 263.61 | -0.64 | 0.08730 | 0.30179 |
| ADRBK1 | 197.48 | 337.77 | -0.77 | 0.09315 | 0.31261 |
| NEU3 | 2.29 | 1.24 | 0.89 | 0.09960 | 0.32530 |
| MLC1 | 8.64 | 5.87 | 0.56 | 0.10790 | 0.33972 |
| CSNK2A1 | 21.57 | 15.03 | 0.52 | 0.11325 | 0.34991 |
| FLNA | 331.91 | 786.60 | -1.24 | 0.11360 | 0.35037 |
| AXIN1 | 21.44 | 31.23 | -0.54 | 0.11570 | 0.35414 |
| PLD2 | 6.32 | 9.01 | -0.51 | 0.12340 | 0.36791 |
| AKAP1 | 5.75 | 4.02 | 0.52 | 0.12575 | 0.37161 |
| PRKACA | 67.25 | 95.79 | -0.51 | 0.12990 | 0.37917 |
| PTRF | 0.24 | 0.42 | -0.84 | 0.15320 | 0.41890 |
| PPP2R1B | 4.85 | 2.91 | 0.74 | 0.15850 | 0.42716 |
| LRP1 | 7.83 | 10.66 | -0.45 | 0.16935 | 0.44474 |
| ITCH | 12.92 | 9.31 | 0.47 | 0.18150 | 0.46252 |
| SQSTM1 | 89.66 | 184.87 | -1.04 | 0.18575 | 0.46775 |
| BMPR1A | 0.18 | 0.36 | -0.98 | 0.20640 | 0.49645 |
| PPP1CA | 175.83 | 310.46 | -0.82 | 0.21185 | 0.50342 |
| PPP2R4 | 38.73 | 53.61 | -0.47 | 0.23440 | 0.53256 |
| HSP90AA1 | 48.94 | 64.49 | -0.40 | 0.24095 | 0.54142 |
| SRC | 10.35 | 14.59 | -0.50 | 0.24960 | 0.55148 |
| TSC2 | 18.25 | 28.33 | -0.63 | 0.25080 | 0.55267 |
| ESR1 | 0.97 | 0.18 | 2.47 | 0.25240 | 0.55432 |
| PLD1 | 1.70 | 1.25 | 0.45 | 0.31005 | 0.61626 |
| ERBB2 | 2.55 | 3.27 | -0.36 | 0.31520 | 0.62210 |
| ABCB1 | 4.34 | 3.22 | 0.43 | 0.31910 | 0.62533 |
| VCP | 73.24 | 95.90 | -0.39 | 0.34570 | 0.65352 |
| ITPR3 | 7.78 | 6.34 | 0.30 | 0.37310 | 0.68110 |
| PTGS2 | 1.55 | 2.01 | -0.38 | 0.37550 | 0.68398 |
| CD40 | 11.48 | 14.00 | -0.29 | 0.44025 | 0.74261 |
| VDAC1 | 38.34 | 32.37 | 0.24 | 0.44280 | 0.74455 |
| BTK | 48.79 | 59.84 | -0.29 | 0.44945 | 0.74885 |
| ACIN1 | 61.67 | 74.95 | -0.28 | 0.47950 | 0.77238 |
| PTPN11 | 8.82 | 4.42 | 1.00 | 0.48640 | 0.77777 |
| FYN | 98.50 | 117.00 | -0.25 | 0.50990 | 0.79514 |
| CSNK2A2 | 8.26 | 7.05 | 0.23 | 0.52330 | 0.80432 |
| AQP3 | 38.07 | 32.91 | 0.21 | 0.53230 | 0.81024 |
| BST1 | 67.51 | 90.54 | -0.42 | 0.56210 | 0.82882 |
| F2R | 3.34 | 2.35 | 0.51 | 0.56665 | 0.83179 |
| TRAF6 | 4.40 | 3.78 | 0.22 | 0.57445 | 0.83793 |
| CAV2 | 0.40 | 0.22 | 0.84 | 0.57660 | 0.83917 |
| MMP14 | 0.83 | 0.68 | 0.29 | 0.58725 | 0.84624 |
| PRNP | 14.65 | 13.16 | 0.15 | 0.64080 | 0.87737 |
| PTEN | 31.97 | 38.18 | -0.26 | 0.65055 | 0.88385 |
| TENC1 | 0.13 | 0.81 | -2.68 | 0.65575 | 0.88688 |
| NOS3 | 9.13 | 10.76 | -0.24 | 0.65830 | 0.88812 |
| PTPN1 | 28.96 | 26.62 | 0.12 | 0.69780 | 0.90802 |
| GRK5 | 13.41 | 14.77 | -0.14 | 0.69835 | 0.90802 |
| PTCH1 | 1.69 | 1.50 | 0.17 | 0.71260 | 0.91458 |
| RHOA | 363.32 | 400.84 | -0.14 | 0.72030 | 0.91935 |
| MCL1 | 279.06 | 256.67 | 0.12 | 0.76550 | 0.93996 |
| INSR | 0.89 | 0.96 | -0.12 | 0.78640 | 0.94601 |
| PPP2CA | 44.57 | 52.81 | -0.24 | 0.78885 | 0.94746 |
| PLAA | 7.22 | 6.79 | 0.09 | 0.83230 | 0.96048 |
| MAPK1 | 41.64 | 38.82 | 0.10 | 0.84280 | 0.96459 |
| NTRK1 | 0.36 | 0.28 | 0.35 | 0.85515 | 0.96783 |
| DNM1 | 0.45 | 0.61 | -0.42 | 0.85835 | 0.96887 |
| PROCR | 0.57 | 1.12 | -0.98 | 0.86225 | 0.97012 |
| CD44 | 145.66 | 140.18 | 0.06 | 0.90635 | 0.98030 |
| PTPRF | 0.51 | 0.53 | -0.05 | 0.92715 | 0.98381 |
| NF1 | 2.04 | 1.80 | 0.18 | 0.94505 | 0.98695 |
| SCP2 | 44.73 | 44.37 | 0.01 | 0.97425 | 0.99300 |
| PDIA3 | 143.94 | 142.13 | 0.02 | 0.98180 | 0.99470 |
